# Clover sign of Crohn's disease

**DOI:** 10.1002/jgh3.12619

**Published:** 2021-07-17

**Authors:** Akira Hokama

**Affiliations:** ^1^ Department of Endoscopy, Graduate School of Medicine University of the Ryukyus Okinawa Japan

**Keywords:** Crohn's disease, enteroenteric fistula, the clover sign

## Abstract

The clover sign is a useful and characteristic radiological feature for diagnosing Crohn's disease with enteroenteric fistulae.

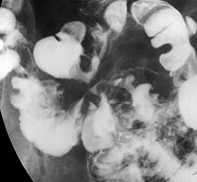

## Introduction

Contrast radiography and computed tomography scans play important roles in the diagnosis and management of Crohn's disease.^1^ Many radiographic signs, including cobblestone sign, string sign, and shell sign, are pathognomonic for Crohn's disease. I highlight another feature that may assist diagnosing enteroenteric fistulae in Crohn's disease.

## Case report

**Figure 1:**
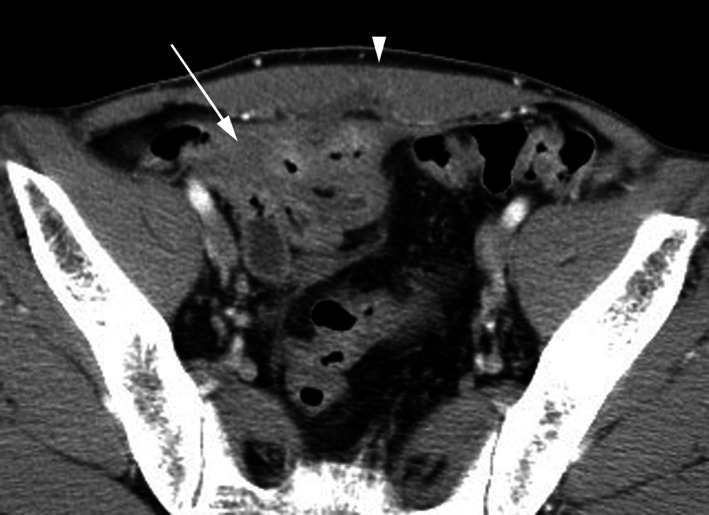
A computed tomography scan revealed the enterocutaneous fistula (arrowhead) and the configuration of mildly dilated bowel loops (arrow).

**Figure 2:**
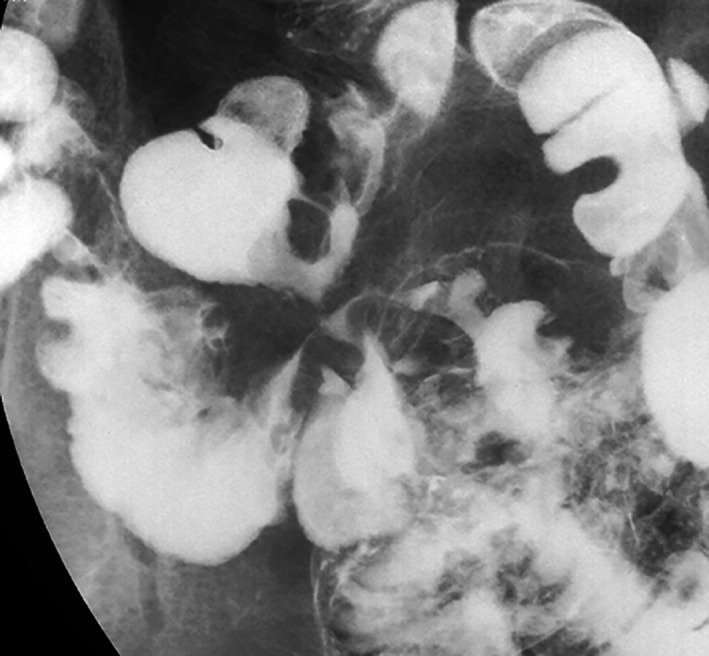
A small bowel follow‐through study showed clover leaves‐like arrangement of the prominent pseudodiverticula that converge to a central point, consistent with the multiple ileoileal fistulae.

In 2007, a 49‐year‐old man with Crohn's disease was investigated because of diarrhea, body weight loss, and an enterocutaneous fistula. His medical history included an ileal resection of ileal stenosis 21 years previously. Physical examination revealed decreased body mass index of 17 kg/m^2^. Laboratory tests showed a white blood cell counts of 6.2 × 10^9^/L, a hemoglobin level of 108 g/L, and a C‐reactive protein of 24.8 mg/L. A computed tomography scan revealed the enterocutaneous fistula and the configuration of mildly dilated bowel loops, suggesting ileoileal fistulae (Fig. 1). A gastrografin follow‐through revealed the prominent pseudodiverticula interconnected by multiple ileoileal fistulae, resembling “the clover leaves” (Fig. 2). As infliximab was not effective for both fistulae, he underwent partial ileotomy and fistulectomy. The patient has been in clinical remission with mesalazine and elemental diet for 14 years.

## Discussion

The clover‐leaf deformity with pseudodiverticulum formation by duodenal ulcer is a well‐known radiographic sign. Another clover sign, clover leaves‐like arrangement of intestines that converge to a central fistula point, is a useful and characteristic radiological feature for diagnosing Crohn's disease with enteroenteric fistulae. The transmural inflammation of Crohn's disease leads to a narrow lumen with impaired distensibility.^1^ The predilection of Crohn's longitudinal ulcers for the mesenteric border can cause marked fibrosis and shortening, which leads to sacculations on the antimesenteric border and forms pseudodiverticula.^2^ As ulcers penetrate deeply, they can also cause fistulous formation.^1^ Combined radiographic appearance of the pseudodiverticula and fistulae resembles the clover leaves.

The small bowel follow‐through study can present the clear detection of whole fistulae in a single image. A similar sign of these lesions detected by magnetic resonance enterography (MRE) is called “the star sign.”^3^ Although MRE has a better visualization of inflammation of the bowel wall along with the fistulous tracts, the clover sign by the small bowel follow‐through study may help direct identification of intestines interconnected by enteroenteric fistulas.
